# Origins of Improved Hardness in Bioinspired Aragonite Precipitated Under Simulated Biologic Conditions

**DOI:** 10.1111/gbi.70060

**Published:** 2026-09-10

**Authors:** Boontharee Chomhom, Gabriela A. Farfan, Samantha Greeves, Kirsty Penkman, Adrian A. Finch, Susan Fitzer, Roland Kröger, Alex Brasier, John Still, Matthieu Clog, Nicola Allison

**Affiliations:** ^1^ School of Earth and Environmental Sciences, University of St Andrews St Andrews UK; ^2^ Scottish Ocean Institute, University of St Andrews St Andrews UK; ^3^ Department of Mineral Sciences National Museum of Natural History, Smithsonian Institution Washington DC USA; ^4^ Department of Chemistry University of York York UK; ^5^ Faculty of Natural Sciences, Institute of Aquaculture University of Stirling Stirling UK; ^6^ School of Physics, Engineering and Technology University of York York UK; ^7^ School of Geosciences University of Aberdeen Aberdeen UK; ^8^ SUERC, University of Glasgow Glasgow UK

**Keywords:** amino acid, aragonite, hardness, nanograin, structure distortion

## Abstract

Biogenic aragonite is a composite of mineral and biomolecules that exhibits superior material properties compared to its inorganic analog. This superiority underpins the success of the bioaragonite structures which support coral reefs and shell fisheries, yet the mechanisms improving the physical resilience of bioaragonite are not well understood. In this study, aragonite is synthesised under chemical conditions which simulate those of calcification sites in marine organisms. Aspartic acid, glutamic acid, and glycine in solution are incorporated into the aragonite at concentrations typically observed in marine biominerals and are observed to significantly improve aragonite Vickers hardness. Aspartic acid is incorporated more efficiently than glutamic acid or glycine, but for comparable amounts of amino acid incorporation, glycine improves aragonite hardness significantly more than either aspartic or glutamic acid. Glycine and glutamic acid incorporation reduce the aragonite grain size, thereby improving resilience to indentation via energy dissipation along grain boundaries. In contrast, aspartic acid also improves hardness on a smaller scale, possibly due to crystal structure distortions. These results suggest that amino acid incorporation influences biogenic aragonite hardness via multiple mechanisms and provide a prototype for exploring the influence of more complicated biomineral proteins.

## 1 Introduction

Multiple marine phyla produce CaCO_3_ structures, e.g., skeletons and shells that provide tissue support and protection. The structures are nanocomposite materials composed of CaCO_3_ interspersed with biomolecules (proteins, lipids, polysaccharides) (Mass et al. 2014). These biominerals exhibit superior material properties (such as increased Vickers hardness and fracture resistance) compared to inorganically precipitated analogs (Huang et al. 2019). Yet, mechanisms underlying the hardening of these biominerals are not well understood.

The hardness of biominerals has been linked to features at the nm and Å scales (Huang et al. 2019; Kakisawa and Sumitomo 2011). On a larger scale, both nacre and coral aragonite are composed of mineral nanograins embedded in an organic matrix (François et al. 2012; Mouritz 2012; Christian and Mahajan 1995; Kakisawa and Sumitomo 2011; Kim et al. 2016; Risan et al. 2018; Borukhin et al. 2012; Pokroy et al. 2004; Iwase and Mori 2020; Deng et al. 2020; Li et al. 2004; Oaki et al. 2006; Cuif and Dauphin 2005; Dauphin et al. 2006; Drake et al. 2020). The organic materials surrounding the nanograins are ductile and can flatten under local stress to absorb energy (Katti et al. 2005). Polycrystallinity also influences material hardness, as grain boundaries provide resistance to dislocation motion and crack propagation (Deng et al. 2020; Deng and Li 2021; Jeong et al. 2022).

On an atomic scale, biomolecules in biogenic aragonite are associated with crystal structure distortions (compared to inorganic aragonites) that could be linked to inter or intracrystalline organic compositions (Pokroy et al. 2004, 2006, 2007; Zolotoyabko and Pokroy 2007). Such defects may inhibit dislocation motion and deformation twinning (Christian and Mahajan 1995). However, to date, no studies have explored how these distortions in bioaragonite correlate with Vickers hardness. Thus, we turn to biocalcite studies that more closely examine the impact of increased crystal dislocations (Kim et al. 2016; Risan et al. 2018; Borukhin et al. 2012) and deformation twinning (Christian and Mahajan 1995) from amino acids (Kim et al. 2016) and proteins (Risan et al. 2018) on increasing Vickers hardness.

This study explores the origin of hardness in bioinspired aragonite produced under the conditions inferred to occur in the calcification media of tropical corals (Castillo Alvarez et al. 2024). Aragonite is synthesised with no amino acids as a control and in the presence of variable concentrations of L‐aspartic acid (Asp), L‐glutamic acid (Glu), and L‐glycine (Gly). These amino acids are important components of biomineralization proteins (Weiner 1979; Cuif et al. 1999; Bédouet et al. 2001; Suzuki et al. 2009; Kellock et al. 2020) and influence CaCO_3_ nucleation, growth, morphology, and structure (Kim et al. 2016; Castillo Alvarez et al. 2024; Picker et al. 2012; Kellock et al. 2022). The incorporation of the amino acids into the aragonite is quantified and the influence of the amino acids on aragonite properties, crystal structure, and morphology is determined by micro‐indentation, powder X‐ray diffraction (pXRD) and scanning electron microscopy (SEM). The data are compared to resolve relationships between aragonite hardness, structure distortions, and crystal nanograin microstructure under conditions that occur in marine calcifiers.

## 2 Methods and Materials

### 2.1 In Vitro Aragonite Precipitation Experiments

**FIGURE 1:**
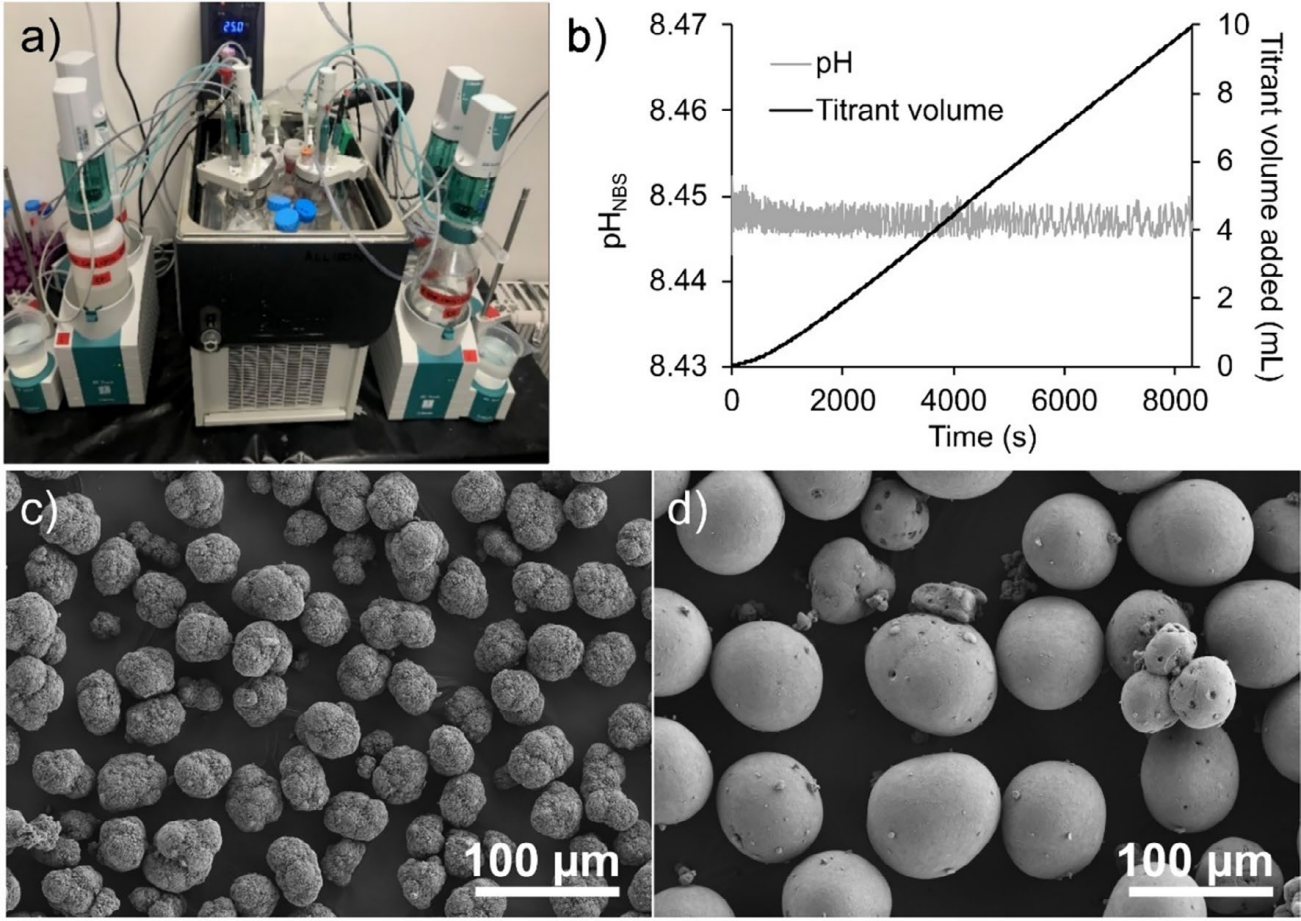
Overview of the study illustrating: (a) pH stat titrators used for aragonite synthesis (Castillo Alvarez et al. 2024; Kellock et al. 2020, 2022), (b) a typical profile of pH control and titrant dosing within each titration, and scanning electron micrographs of aragonite particles formed (c) with no amino acids and (d) with 5 mM aspartic acid.

Aragonite was synthesised in vitro using a pH‐stat titrator which doses both Na_2_CO_3_ and CaCl_2_ to maintain a constant pH [CO_3_^2−^], [Ca^2+^] and thereby aragonite saturation state (Ω) during precipitation (Figure 1) (Kellock et al. 2022). Aragonite was precipitated from artificial seawater (Millero 2013) with no amino acid and in the presence of 1 μM, 100 μM and 5 mM of L‐Asp, L‐Glu or L‐Gly (Signa‐Aldrich, all > 99% purity). These are common amino acids in coral skeletons and mollusk shells (Weiner 1979; Cuif et al. 1999; Kellock et al. 2020). The high [Mg^2+^] of modern seawater favours the precipitation of aragonite over calcite (Berner 1975). In total, we ran 10 precipitation experiments with each experiment lasting up to approximately 2 weeks (Table S1). The apparatus was maintained in a water bath at 25°C for all experiments. The artificial seawater had total alkalinity 2352 μmol kg^−1^ and salinity 34.1 and was bubbled with atmospheric air (with a [CO_2_] of ~410 ppm) to reach CO_2_ equilibrium. 340 mL of this seawater was measured into the reaction vessel, a high‐density polyethene (HDPE) beaker with a total volume of ~500 mL. The dissolved inorganic carbon (DIC) of the solution was increased to 4000 μmol kg^−1^ by adding 0.6 M Na_2_CO_3_. If used, pre‐weighed amino acid was dissolved in ≤ 1 mL seawater and pipetted into the reaction vessel to give the required amino acid concentration. The amino acid was added, if used, and the solution was immediately adjusted to pH 8.448 (NBS scale) with the addition of 2 M HCl. This generates a solution in equilibrium with atmospheric CO_2_. The exact conditions and dimensions of the coral calcification site are unknown but our experimental manipulations produce a precipitating solution with pH and Ω_Ar_ similar to those observed in coral calcification media (Sevilgen et al. 2019).

After adjusting solution pH_NBS_ to 8.448, 200 mg of an aragonite seed was added to provide a growth surface for the aragonite. The ~15–20 μm‐diameter seeds were produced by grinding synthetic aragonite precipitated from seawater at Ω_Ar_ = 11 in an agate ball mill (Allison et al. 2024). The reaction vessel was capped with an ethylene tetrafluoroethylene lid with ports through which the pH/temperature sensor, a propeller stirrer, a gas tube and the 2 titrant dosing tubes were inserted. The pH of the solution was constantly monitored using a high precision pH/temperature sensor (Metrohm Aquatrode Pt1000) calibrated weekly with NIST buffers. CaCO_3_ precipitation reduces seawater pH and this decrease triggers the addition of equal volumes of 2 titrants (0.6 M Na_2_CO_3_ and 0.6 M CaCl_2_ and SrCl_2_ in a 99:1 ratio) from an adapted Metrohm Titrando 902 titrator to replace the ions consumed in precipitation. The second titrant contains a mixture of Ca and Sr. to reflect the substitution of Sr^2+^ for Ca^2+^ into the aragonite (Finch et al. 2003). The precipitation continued until 10 mL of each titrant was added. The aragonite in the vessel (initial seed plus precipitate) was collected by vacuum filtration onto a 0.2 μm polyether sulfone (PES) filter. The entire precipitation was repeated using fresh artificial seawater and using the aragonite collected from the previous titration as a seed. This enabled aragonite to grow sequentially onto the original seed, producing large particles (typically 30–50 μm in diameter). This sequence was repeated 8 times to produce aragonite in the presence of no amino acid or with 100 μM and 5 mM of each of the amino acids. The sequence was repeated 6 times to produce aragonite in the presence of 1 μM amino acids. After the final titration, the aragonite was collected onto a filter as before, rinsed with deionised water and dried at 40°C overnight. The procedure resulted in the precipitation of ~3.6 to 4.8 g of aragonite in vitro (depending on the number of sequence repeats). The seed, provided for aragonite growth, contributed 6% or less of the final solid recovered. We consider this amount to have an insignificant influence on the amino acid composition and pXRD of the final sample. We used Raman spectroscopy (Kellock et al. 2022) to confirm that aragonite was the sole precipitate polymorph. We collected at least 12 Raman spectra from each precipitate and found that lattice mode peaks at 100–250 cm^−1^ in all spectra were consistent with aragonite (Figure S3) with no evidence of the features associated with calcite or amorphous calcium carbonate (DeCarlo 2018). Further, we do not observe wider ν_1_ modes that would suggest the presence of ACC, as has been observed in previous precipitation studies of calcite + ACC (Fang et al. 2023). We also confirmed that all precipitates were aragonite via XRD, for which all patterns lacked a calcite (104) peak (Figure S2). As ACC is X‐ray amorphous, it does not produce any reflections in XRD, and we did not observe any clear presence of ACC phases in the background of our XRD patterns (Figure S2). Future studies may perform more targeted time‐series studies to find any shorter‐lived ACC phases in precipitates using Raman spectroscopy and XRD.

### 2.2 Amino Acid Analysis

The amino acid composition of the aragonite was determined for all precipitated samples, including the controls, following the methods of Tomiak et al., 2013. < 20 mg of powdered aragonite was accurately weighed into a plastic microcentrifuge tube and bleached using 50 μL 12% sodium hypochlorite (NaOCl) per mg to isolate the intracrystalline fraction of amino acids. This chemical treatment removes any organic materials which are not intimately embedded in the aragonite mineral. We define the amino acid material remaining after treatment as ‘intracrystalline’, although we do not assume that the amino acid has substituted into the aragonite lattice. After 48 h, samples were sequentially rinsed with 18.2 MΩ deionised water and methanol and left to air‐dry. < 10 mg of each dried sample was accurately weighed into a 2 mL sterile glass vial, demineralized in 2 M HCl (10 μL/mg) and spun to dryness in a centrifugal evaporator. Samples were rehydrated in a solution containing 0.01 mM L‐homoarginine (as an internal standard) and analyzed using an Agilent 1100 HPLC with a fluorescence detector. All samples were run in duplicate alongside standards and blanks.

### 2.3 Hardness Measurement and Calculations

Hardness was measured using a Leco Vickers LM 248AT micro indentation hardness tester equipped with a pyramid diamond indenter. Aragonite particles were embedded in epoxy resin (Epofix, Struers Ltd), and polished using silicon carbide papers (up to 4000 grades, lubricated with water) and 3 and 0.25 μm diamond suspensions to produce particle cross sections. The indenter was impressed into polished cross sections of the sample (not acid etched) using a mass of 20 g for a period of 10 s. The measurements were made at the edges of the particles where the aragonite precipitated in the experiment was located and not at the center of the particle samples where the original CaCO_3_ seed was located. After indentation, the cross‐sections were gold‐coated and imaged by SEM using a JEOL JSM IT200. SEM micrographs were imported into ImageJ, and the two diagonal lengths of the indent were measured. The Vickers hardness number (HV) was calculated by the ratio of the force applied by the indenter and the surface areas of the final indent by using the equation below (Fitzer et al. 2019):

$$\mathrm{HV}=\left(0.1891^{*}\;F\right)/\left(\text{indent area}\;\left(d\right),{\mathrm{mm}}^{2}\right)$$

where *F* is Vickers hardness, *F* is applied force, and indent area (d) is an average dimension of the indent in mm. HV was converted to GPa (gigapascals) by multiplying by 0.009807. Ten to 16 indents were made on each particle population and only one indent was made on each particle. Variations in hardness between the aragonite samples precipitated with amino acid and the control are identified using ANOVA followed by Tukey's pairwise comparison.

### 2.4 pXRD and Rietveld Refinements

XRD analysis of the synthetic aragonite powders was performed using a Rigaku D/Max Rapid micro‐X‐ray diffractometer with Debye‐Sherrer geometry using Mo Kα radiation (*λ* = 0.71069 Å) at 50 kV and 40 mV to capture X‐ray scattering from 4 to 44 degrees 2θ. XRD was performed on the control aragonite and samples precipitated with 100 μM and 5 mM of each amino acid. All powder samples were mounted in a 1 mm inner‐diameter polyamide capillary, capped with clay. Data were collected at three positions along each capillary (top, middle, and bottom) for 20 min each with omega fixed at 0° and phi rotating at 1°/s. Two capillary tubes were prepared and analysed for each sample, resulting in 6 replicate analyses. 2DP software was used to background‐correct and integrate (80–400 β) image plate data into intensity versus 2θ patterns.

Whole pattern Rietveld refinement analyses were performed using GSAS II with a Chebyschev polynomial background (Toby and Von Dreele 2013). We modelled the aragonite using a previously published geological aragonite structure (Antao and Hassan 2009). The unit cell axis lengths and volumes were compared between samples by Kruskal‐Wallis followed by a Bonferroni corrected Dunn's post hoc test. The error in each sample is represented by the standard deviation between all six sample replicates.

### 2.5 Scanning Electron Microscopy of Particle Microstructure

Samples were imaged using a Carl Zeiss GeminiSEM 300—high‐resolution Field Emission Scanning Electron Microscope (FESEM) with SE (secondary electron), BSE (backscattered electron), and CL (cathodoluminescence) detectors. An accelerating voltage of 2.5 keV and an SE2 detector was used for low magnification images (<x1000), and an accelerating voltage of 2.5 keV and an InLens secondary electron detector were used for magnifications ≥ ×1000. Powder samples were mounted on double carbon tape onto stubs and carbon‐coated under vacuum (Quorum K950 carbon coater), rotating samples 90° between coats to ensure full coverage. The internal morphology of the aragonite particles was exposed by embedding the samples in epoxy resin and polishing a cross‐section through the particles. Particle cross sections were carbon coated and imaged as before. The untreated cross‐section surfaces are observed to be flat and featureless (Figure S1a), so the particle microstructure was highlighted by etching the cross‐sections with 0.5% acetic acid for 30 s prior to carbon coating (Figure S1b). Acid etching was only used for imaging of the cross‐section microstructure and was not used prior to indentation, XRD, or amino acid analysis.

## 3 Results

### 3.1 Amino Acid Incorporation

**FIGURE 2:**
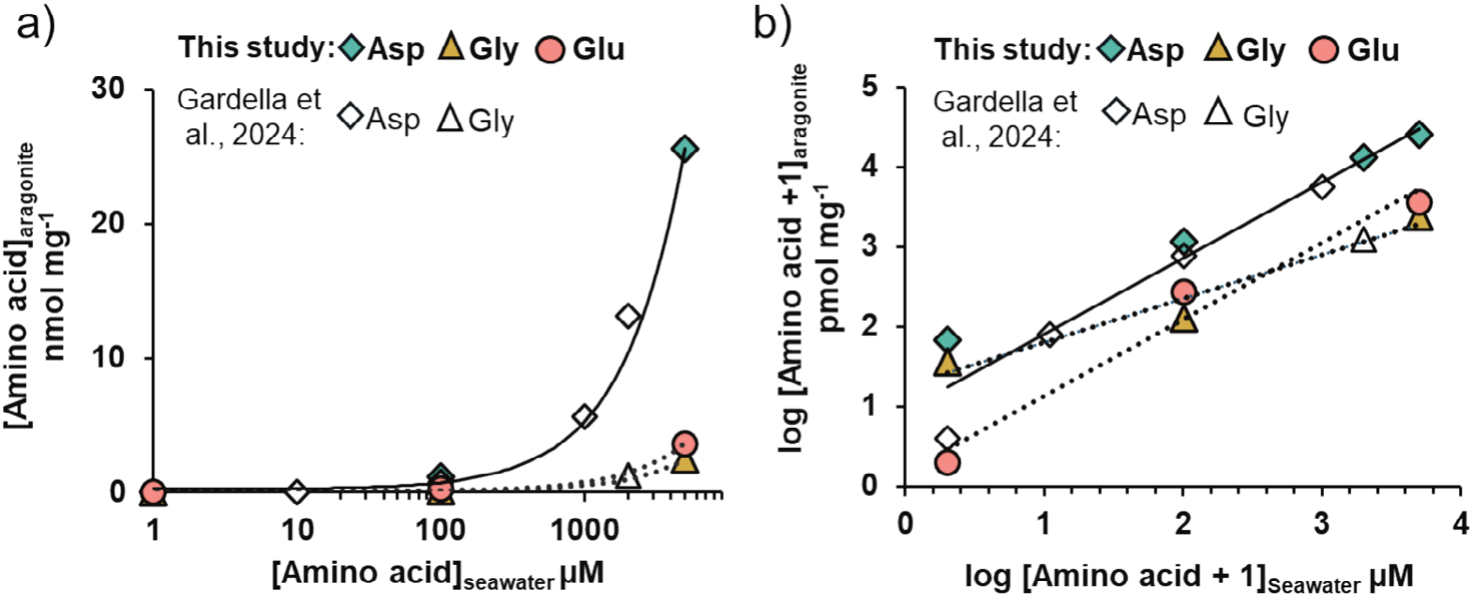
Amino acid incorporation in aragonite precipitation experiments at a range of [amino acid]_seawater_ plotted as (a) normal‐log plot and (b) log–log plot. Values are presented in Table S1. Errors in amino acid concentration measurements in aragonite are smaller than the symbols. Data from this study are compared to measurements by Gardella et al. (2024) of Asp and Gly incorporation in aragonite under similar conditions (Table S1).

All the amino acids are incorporated into aragonite in approximately logarithmic relationships between the concentration in seawater ([amino acid]_seawater_) and the concentration occluded in the aragonite ([amino acid]_aragonite_) (Figure 2a,b, Table S1). For log plots, we plot log [amino acid+1]_seawater_ to enable us to plot the control on the same graph. These results agree with a previous study on Asp and Gly (Gardella et al. 2024). Glu and Gly are incorporated much less efficiently than Asp at comparable seawater concentrations, despite Asp and Glu having very similar physicochemical properties (Fang et al. 2023). The resulting concentration of Asp in aragonite in this study follows similar trends as Gardella et al. (2024) (Table S1) for [Asp]_seawater_ = 1 and 100 μM (Figure 2).

Assuming that any amino acid not incorporated into the aragonite remains in solution, the [amino acid]_seawater_ usually decreases by < 2% during each titration. The exceptions are the precipitations with 1 μM Asp and 1 μM Gly in which [amino acid]_seawater_ decreases by 12% and 6%, respectively. These decreases are small in comparison with the range of concentrations tested.

### 3.2 Amino Acid Effects on Aragonite Morphology at the μm and Nm Scale

**FIGURE 3:**
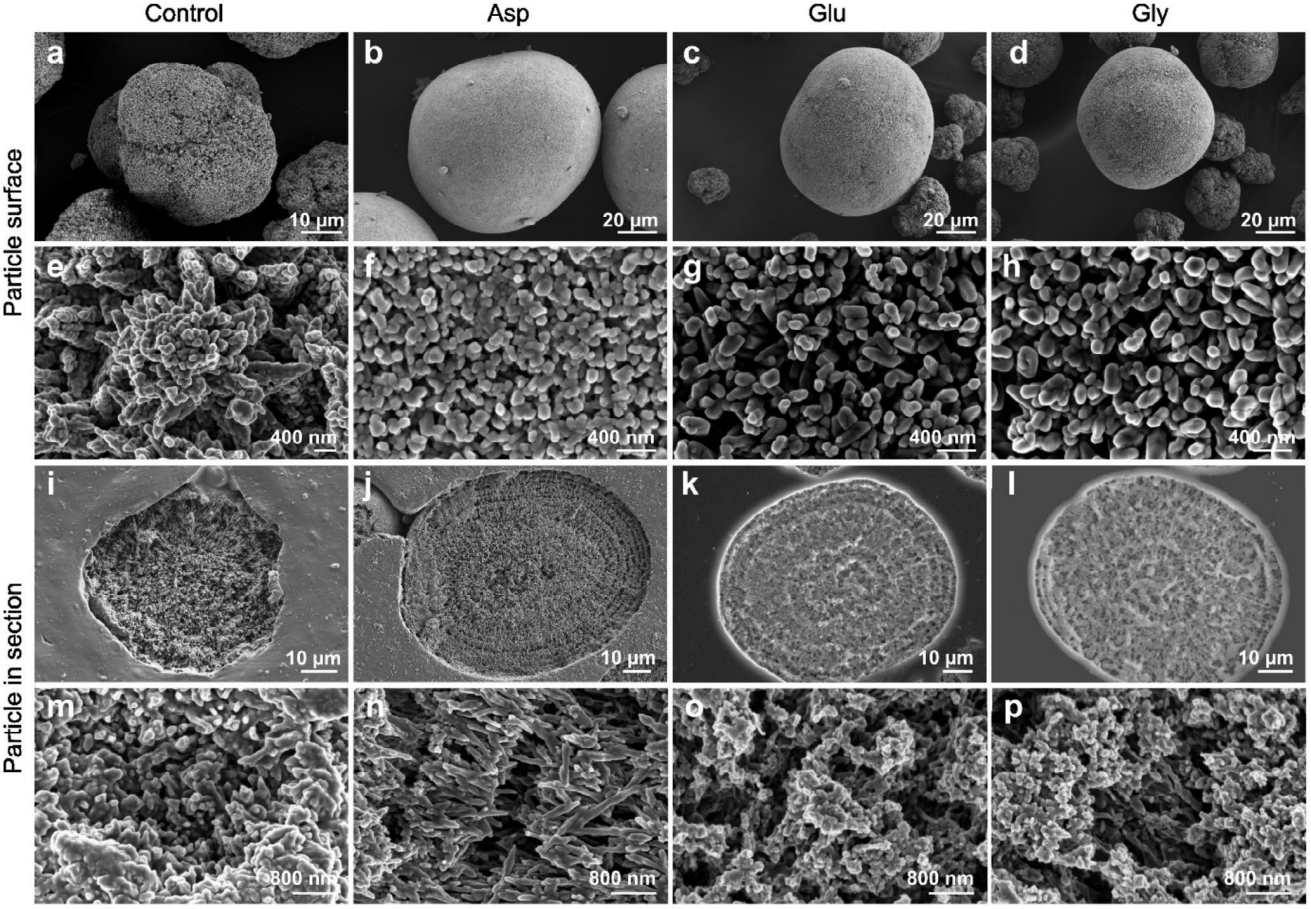
Morphology of aragonite precipitated with no amino acid (control), Asp, Glu, and Gly (all 5 mM). Scanning electron micrographs of (a–d) particles, (e–h) particle surfaces, (i–l) particle cross‐sections, and (m–p) internal morphology.

SEM observations indicate how amino acid incorporation alters aragonite morphology (Figure 3). Aragonite particles precipitated with no amino acid (control) are irregular in shape (Figure 3a) and the surfaces are dominated by crystals exhibiting an acicular pyramidal morphology (Figure 3e). The crystals are composed of nanoparticles typically 100–200 nm along each dimension, as in previous observations of aragonite formed under similar conditions (Castillo Alvarez et al. 2024).

Aragonite particles precipitated with Asp, Glu, and Gly (Figure 3b–d) are markedly different from the control (Figure 3a). They are more spherical with smoother surfaces and a much finer‐grained surface morphology. Scanning electron micrographs of the particle surfaces record the morphology of the aragonite crystals looking down the *c*‐axes of the crystals. Previous observations of aragonite crystal formation have determined that aragonite may grow 10 times faster along the *c*‐axis than along the *a*‐axis (Sun et al. 2017), which is consistent with the spherulitic growth of the particles grown with the amino acids.

In cross section, all the etched particles precipitated with amino acids exhibit a ring structure consisting of concentric zones of higher porosity up to 700 nm in width which are separated by zones of aragonite crystals up to 2 μm wide (Figure 3j–I; Figure S1b). Observations were focused on particles where rings occur close to the innermost areas of the particle as well as towards the particle outside, i.e., demonstrating that the cross‐section runs through the approximate center of the particle and that crystal growth along the *c*‐axis is parallel to the section surface. The higher porosity rings indicate the dissolution of material during the etch, likely indicating where higher concentrations of organics and/or smaller crystallites occur (Janiszewska et al. 2018; Fang et al. 2024). Although the etch removes some of the aragonite from the sample, it is clear that crystal morphology varies between samples after etching. Between the higher porosity rings, the aragonite precipitated with Asp has grains which are typically up to ~1 μm in length (along the *c*‐axis) and ~100 nm in width (Figure 3n). The particles precipitated with Glu and Gly are composed of smaller grains which are equant and similar in size in both dimensions (from 50 to 200 nm) (Figure 3o,p). Compared to the aragonites precipitated with amino acids, the concentric rings are less visible in the control aragonite precipitated with no amino acid and the interior grains have larger dimensions of up to ~200 nm (Figure 3m), as observed in the particle surface micrographs (Figure 3e).

### 3.3 Amino Acid Incorporation and Crystal Lattice Distortion

**FIGURE 4:**
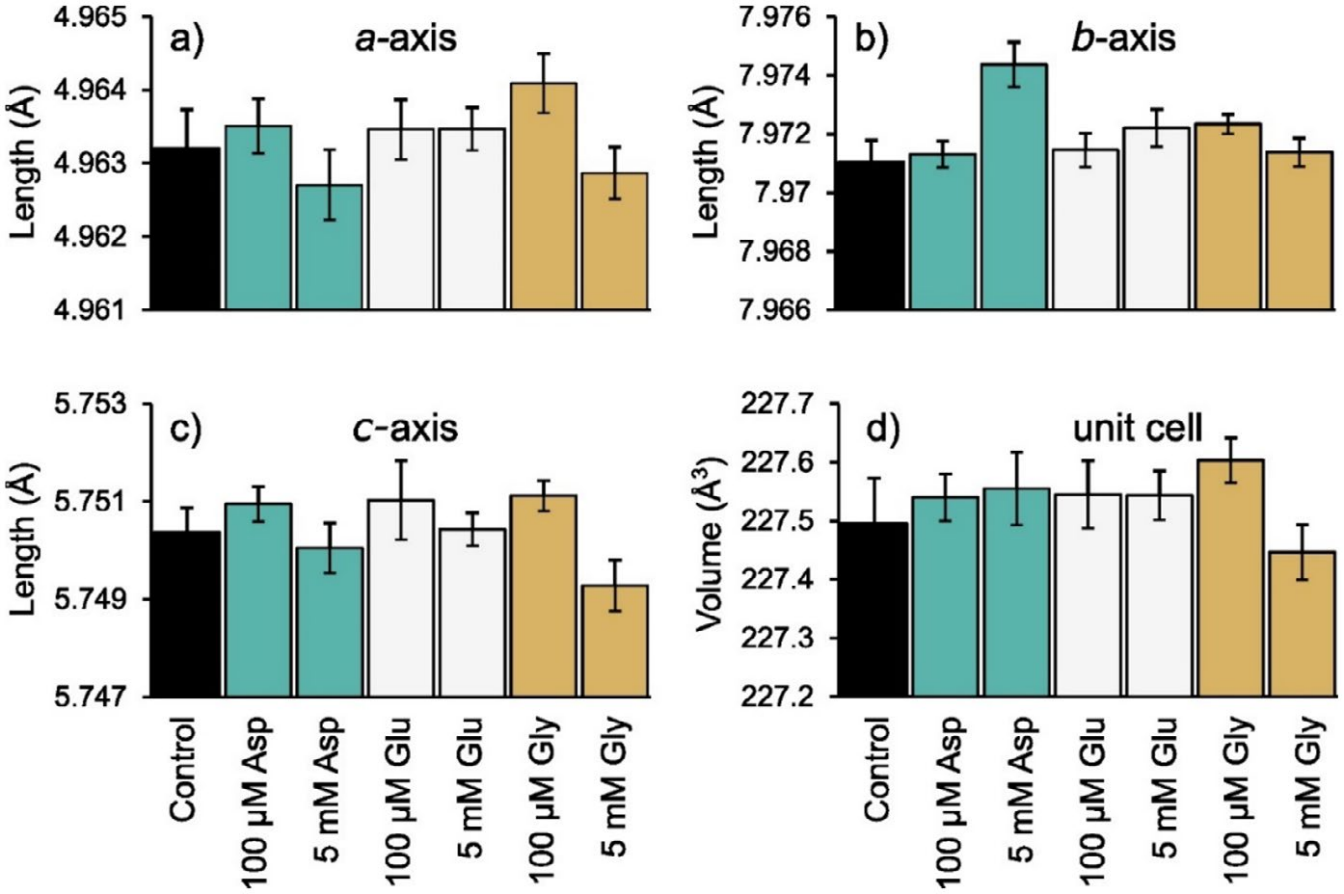
Unit cell parameters of aragonite precipitated with varying [amino acid]_seawater_. (a) *a*‐axis length (Å), (b) *b*‐axis length (Å), (c) *c*‐axis length (Å), and (d) unit cell volume (Å^3^). Values are means and 95% confidence limits of 6 measurements.

To explore the relationship between aragonite hardness and crystal structure, pXRD, analyzed by whole‐pattern Rietveld refinement modelling, was applied to identify the dimensions of the unit cell in each of the aragonite samples (Figure 4, Table S3). Previous studies on bioaragonite crystallography have demonstrated that this technique is useful for measuring anisotropic sub‐angstrom shifts in aragonite unit cells across different organisms and environmental treatments (Pokroy et al. 2004, 2006, 2007; Farfan et al. 2021, 2018). Kruskal–Wallis followed by a Bonferroni corrected Dunn's post hoc test was used to compare the lattice parameters between different groups (Table S4). The *b*‐axis in aragonite precipitated with 5 mM Asp or Glu is significantly longer (*p*_Asp_ < 0.0001; *p*_Glu_ = 0.019) than in the aragonite control produced with no amino acid (Figure 4b; Table S4). In contrast, the *c*‐axis length of the 5 mM Gly experiment is significantly shorter than the control (*p* = 0.038; Table S4). Upon decreasing the concentration of glycine to 100 μM, *a*‐ and *b*‐axis lengths become significantly shorter than the control (*p* < 0.01). Comparing amino acid experiments to each other, we observe that the *a*‐axis of aragonite precipitated with 100 μM Gly is significantly longer than in the sample precipitated with 5 mM Gly, while the opposite is true for the *c*‐axis (Table S4).

**FIGURE 5:**
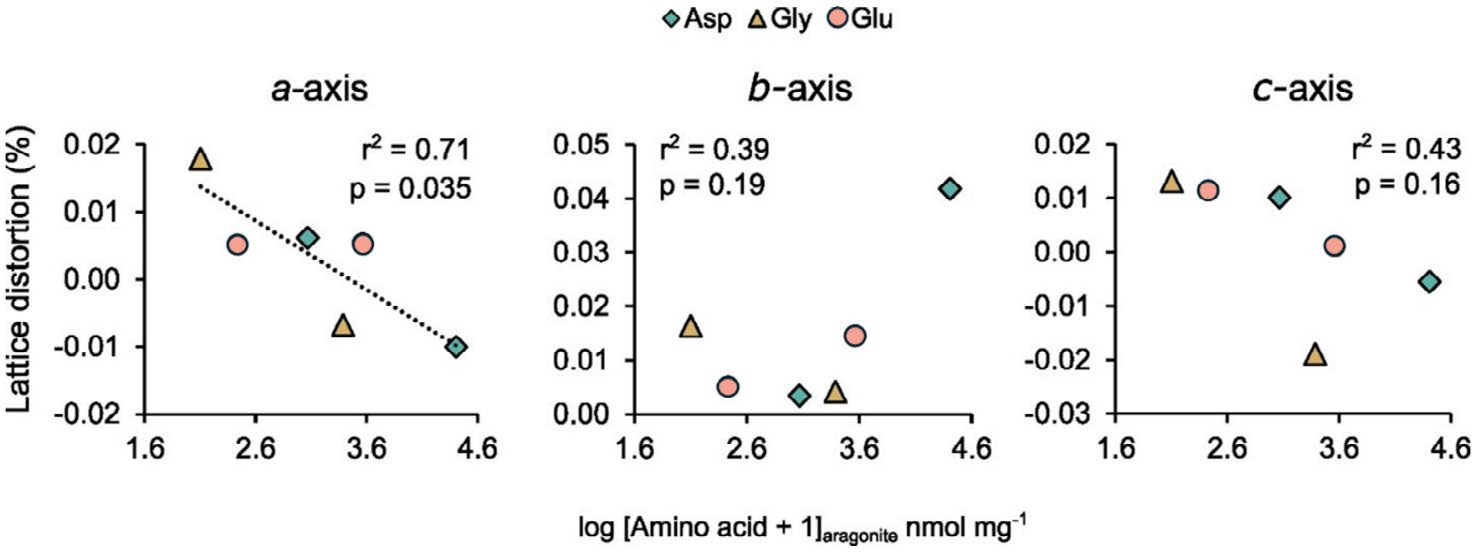
Lattice distortions as a function of [amino acid]_aragonite_. Lattice distortions are calculated as (sample axis length—control axis length)/control axis length as in Borukhin et al. (2012). Coefficients of determination (*r* ^2^) and *p* values are shown. The relation is significant in the *a*‐axis, and a linear trendline is plotted. Errors presented as standard deviations (*n* = 6) are smaller than the symbols.

Unit cell *a*‐axis length versus amino acid incorporation in aragonite shows a significant inverse relationship (Figure 5a). Unit cell axis lengths increased above that of the control (lattice distortion % = 0) at low [amino acid]_aragonite_ but decreased at higher [amino acid]_aragonite_. Relationships between crystal structure distortions and [amino acid]_aragonite_ are not significant for the *b*‐ and *c*‐axes (Figure 5b,c).

### 3.4 Incorporation of Amino Acids on the Hardness of Aragonite

**FIGURE 6:**
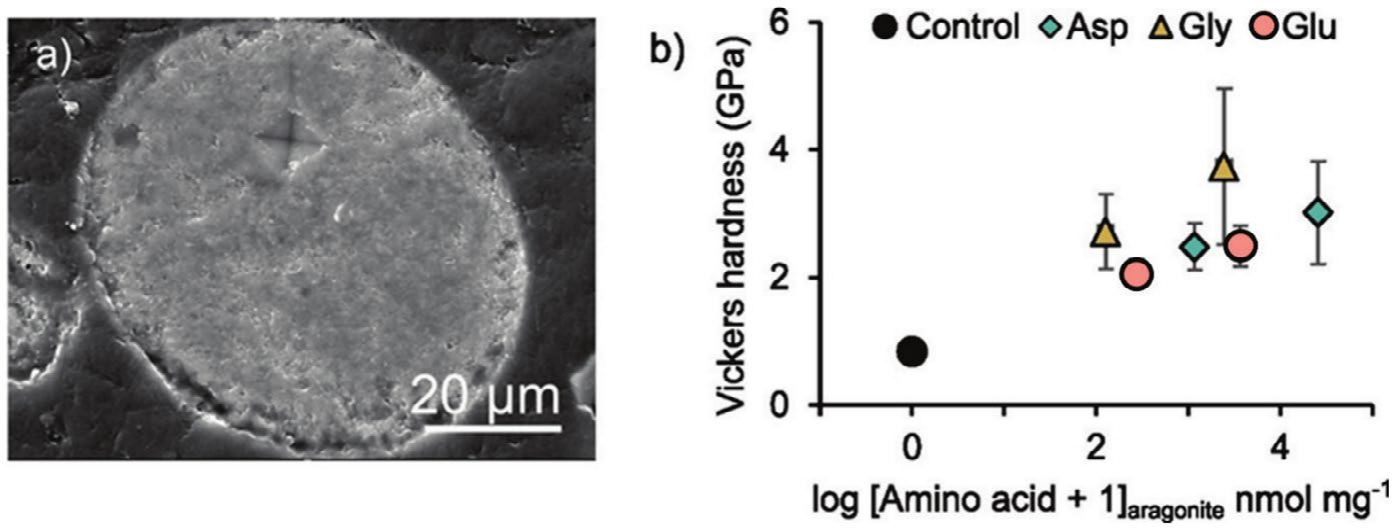
(a) Scanning electron micrograph of a sample indent and (b) aragonite Vickers hardness as a function of [amino acid]_aragonite_ for each of the sets of particles. Values are means from 10 to 16 indents on each particle population and error bars are 95% confidence limits calculated as *t* × *σ*/sqrt(*n*). The error for the control is smaller than the symbol. All Vickers hardness raw data is available in the Supporting Information.

Vickers hardness was analysed by micro‐indentation, averaging 10–16 hardness measurements over particles within each control and amino acid treatment (Table S2). The indents in the present study are located on unetched cross‐sections cut through the center of each spherulite particle to minimize orientation effects (Figure 6a) (Kunitake et al. 2013). The amino acids significantly increase the hardness of the aragonite compared to the control particles (Figure 6b, Table 1, one‐way ANOVA *p* = 7.4 × 10^−5^, followed by Tukey's pairwise comparison, *p* ≤ 0.05). Significant positive linear relationships are observed between the [amino acid]_aragonite_ and hardness for Asp and Glu (Figure 4b, *r* ^2^ = 0.99, *p* = 0.00047, *n* = 5). Gly shows an even stronger relationship with increasing Vickers hardness, despite its relatively lower aragonite incorporation compared to Asp (Figure 4b, *r* ^2^ = 1.00, *p* = 0.019, *n* = 3). These relationships are significantly different between the acidic amino acids and Gly (*p* = 0.027, one‐way ANCOVA). Put another way, both Asp and Glu have similar effects on aragonite hardness for each amino acid molecule incorporated into the aragonite, but incorporation of Gly has a greater effect on hardness than the acidic amino acids, molecule for molecule.

**TABLE 1:** Summary of Vickers hardness measurements across aragonites precipitated at varying [amino acid]_seawater_. *p* values were calculated by one‐way ANOVA followed by Tukey's pairwise comparison. Significant values (*p* ≤ 0.05) are highlighted in bold. The errors are represented as the ± of the SD of multiple Vickers hardness measurements (*n*). For more details, see Table S2.

	Control (no amino acids)	100 μM Asp	5 mM Asp	100 μM Glu	5 mM Glu	100 μM Gly	5 mM Gly
Vickers hardness (GPa)	0.84 ± 0.17	2.53 ± 0.67	3.02 ± 2.3	1.99 ± 0.40	2.36 ± 0.68	2.91 ± 1.0	3.43 ± 1.9
*n*	10	16	10	15	16	14	11
*p*	—	**0.024**	**0.0031**	0.20	**0.023**	**0.0076**	**1.4 × 10** ^**−5**^

## 4 Discussion

Our study demonstrates that Asp, Glu, and Gly are incorporated into aragonite under conditions analogous to those of tropical coral calcification media (Kellock et al. 2022) and that there is good agreement in [Asp]_aragonite_ at [Asp]_seawater_ of 1 and 100 μM between our study and Gardella et al. (2024) (Figure 2). All amino acids significantly improve aragonite hardness (Figure 6). Asp is incorporated more efficiently into aragonite from solution than Glu or Gly (Figure 2), but for comparable amounts of amino acid incorporation, Gly improves aragonite hardness significantly more than either Asp or Glu (Figure 6). Here we explore the multiple mechanisms that may be at play, which allow amino acid incorporations to increase Vickers hardness in biomineral contexts.

### 4.1 Micron‐ to Nano‐Scale Mechanisms That Influence Vickers Hardness

Biomolecule incorporation into CaCO_3_ may improve mineral hardness through a range of mechanisms (Kim et al. 2016; Deng et al. 2020; Katti et al. 2005; Deng and Li 2021; Jeong et al. 2022). Material properties, such as yield stress and flow stress, are inversely related to grain size for materials with grain sizes ranging from ~20 nm to hundreds of μm (the Hall–Petch relationship) (Hansen 2004), as grain boundaries prevent sliding and rotation of grains under compression (Deng and Li 2021). For example, aragonite polycrystallinity improves aragonite resistance to indentation (Kakisawa and Sumitomo 2011; Deng and Li 2021). For even smaller grains, size is positively related to both hardness (Jeong et al. 2022; Moynihan et al. 2021) and compressive elastic moduli (Moynihan et al. 2021; Luo et al. 2023), due to the grain size effect. Our scanning electron micrographs indicate that the grain sizes observed within the present study fall within the range of the Hall–Petch relationship. Particles produced with Gly and Glu have smaller grains compared to the aragonite precipitated with no amino acids or with Asp. The higher abundance of grain boundaries in the aragonite particles produced with Gly and Glu contributes to the enhanced hardness of these particles.

In addition to grain sizes, amino acid incorporations also influence aragonite grain morphology, which may impact Vickers hardness. Previous studies show that amino acids affect the morphology of both calcite and aragonite crystals (Castillo Alvarez et al. 2024; Gardella et al. 2024; Orme et al. 2001; Fang et al. 2023; Xie et al. 2005; Elhadj, De Yoreo, et al. 2006). Amino acids bind preferentially to specific CaCO_3_ crystal faces, impeding growth on the faces to which the amino acid adsorbs whilst CaCO_3_ growth continues on the other faces (Wan et al. 2024; Elhadj, Salter, et al. 2006). In this study, Gly and Glu incorporation generates the smallest grains with approximately equal dimensions (Figure 3o,p). In contrast, Asp incorporation leads to acicular needle morphologies (Figure 3m). This could be tied to how Asp becomes incorporated in the precipitates. Interestingly, Asp has the highest incorporation efficiency of the measured amino acids into the aragonite crystal structure, yet the relatively lower impact on Vickers hardness when compared to equal incorporations of Gly, which produce smaller, equant grains. This higher impact of glycine incorporation on Vickers hardness by decreasing grain sizes suggests that grain boundary energy dissipation is likely the stronger mechanism for increasing Vickers hardness in amino acid‐bound aragonite.

### 4.2 Atomic‐Scale Influences on Vickers Hardness

On an atomic scale, multiple studies indicate that biomolecule inclusion significantly alters the unit cell parameters of calcite and aragonite (Kim et al. 2016; Pokroy et al. 2004, 2006; Iwase and Mori 2020). In addition, the substitution of minor or trace element ions for the mineral host ions (Ca^2+^ or CO_3_^2−^) may also influence lattice parameters if the substitutions inflate or deflate the crystal structure (Pokroy et al. 2006; Finch and Allison 2007). This may occur if amino acids complex host ions, e.g., Ca^2+^, altering the CaCO_3_ geochemistry (Finch and Allison 2007). Lattice distortions are inferred to inhibit dislocation motion and deformation twinning, thereby improving hardness. Asp incorporation significantly increased the length of the aragonite *b*‐axis in the high [Asp] treatment in our study, although hardness was improved by all amino acids even when lattice parameters were not affected (Figures 4 and 5). Although aragonite precipitated with Gly has a greater hardness than aragonite precipitated with Asp for comparable amounts of amino acid incorporation, there is no evidence in the pXRD data to indicate different effects of the two amino acids on the aragonite structure. We also note that the [Asp]_aragonite_ and [Gly]_aragonite_ associated with significant lattice distortion (i.e., 25.6 and 2.44 nmol mg^−1^) are considerably higher than that observed in coral and mollusc samples (see Section 4.4).

### 4.3 Comparisons to Vickers Hardness Studies of Biocalcite

In a previous study, the amino acids, e.g., Asp and Gly were inferred to harden the calcite by serving as obstacles to dislocation motion (Kim et al. 2016). The cutting force required to overcome these obstacles is equivalent to the force required to shear covalent bonds in the backbone of these biomolecules and is essentially the same for both amino acids (Kim et al. 2016). More recently, density functional theory (DFT) calculations (Wan et al. 2024) indicate that the incorporation of amino acids in calcite may generate new C‐C covalent bonds between amino acid molecules, either spontaneously (for Asp) or during mineral compression (for Gly). During CaCO_3_ shear, the predominant alteration is the breakage and reformation of the Ca‐O bonds connecting Ca^2+^ and CO_3_^2−^ groups. The C‐C covalent bonds between amino acid molecules are stronger than the ionic calcite Ca‐O bonds and therefore increase material hardness (Wan et al. 2024). Wan et al. (2024) used the elastic constants from the DFT calculations to estimate that Gly incorporation increased calcite hardness, while Asp decreased hardness relative to inorganic calcite. Wan et al. (2024) suggest that Asp and Glu have markedly different effects on calcite shear properties. Thus, modelling (Wan et al. 2024), experimental (Kim et al. 2016) and this study observations disagree, especially when we compare these calcite studies to our aragonite study where we observe that both of these amino acids increase hardness compared to the control. Material properties also differ between crystal planes in both experimental (Kunitake et al. 2013; Lemke et al. 2021) and modelling studies (Wan et al. 2024), reflecting the orientation of bonds within the interface region. Even so, further work is required to resolve whether amino acids are incorporated into calcite and aragonite as single molecules (Kim et al. 2016) or in paired molecules (Wan et al. 2024) and how amino acids are positioned with respect to the unit cell.

In contrast to the calcite studies (Kim et al. 2016; Wan et al. 2024), the grain size and crystal orientation are key factors controlling the micromechanical properties in *Porites* skeleton (Moynihan et al. 2021). Our Vickers hardness observations in aragonite show that incorporation of different amounts of Asp and Gly had contrasting effects on aragonite hardness. It is clear that amino acid incorporation strengthens aragonite. However, the offset in the hardness of aragonite produced with Gly compared to aragonite produced with Asp or Glu indicates that improved hardness in aragonite is not solely attributable to amino acid shearing. Rather, smaller grain size and lattice distortion are likely more significant drivers for hardness in aragonite.

### 4.4 In a Biomineral Context

The [amino acid]_aragonite_ observed in some of the samples synthesised here is comparable to those of biogenic CaCO_3_ samples, indicating that our results are relevant to marine biominerals. Reverse phase high‐performance liquid chromatography (RP‐HPLC) analyses of biogenic CaCO_3_ measure both combined aspartic acid and asparagine (Asx) and combined glutamic acid and glutamine (Glx), as asparagine and glutamine undergo deamination during the preparative steps. Intra‐crystalline [Asx], [Glx], and [Gly] range from 0.3 to 1.6, 0.01 to 0.3, and 0.1 to 0.9 nmol mg^−1^ respectively in modern coral skeletons (Kellock et al. 2020; Allison et al. 2024) and 0.3 to 1.2, 0.3 to 0.6, and 0.1 to 1.7 nmol mg^−1^ in mollusk shells (Penkman et al. 2008). Asparagine and glutamine contribute < 10% of the Asx and Glx in the coral skeletal acidic amino acid rich protein (CARP3) (Mass et al. 2013), indicating that coral skeletal Asx and Glx predominantly reflect Asp and Glu. In the present study, Asp and Glu are incorporated into the synthetic aragonite from amino acid solutions of 100 μM, at comparable concentrations to the biogenic aragonites (Figure 2). Gly is incorporated into synthetic aragonite at concentrations which are comparable to biogenic aragonites from solutions of 100 μM (figure 2, this study) to 2000 μM (Gardella et al. 2024). The Vickers hardness of the synthetic aragonite particles formed from solutions of 100 μM amino acids (2.1–2.7 GPa) is considerably higher than in the control particles (0.8 GPa) and is comparable to scleractinian corals (1.3–5.1 GPa) (Pasquini et al. 2015; Omer et al. 2020; Carrasco‐Pena et al. 2020; Moynihan et al. 2021; Tan et al. 2023).

Our study does not resolve the mechanism of amino acid incorporation into aragonite, but we explore likely scenarios here. At pH 8.4 (the precipitation fluid pH in this study), all the amino acids have a protonated amine group, NH_3_^+^, and a deprotonated α‐carboxylate group, COO^−^ (Lide 2004), while the acidic side chains (COO^−^) of Asp and Glu are also deprotonated (Voet et al. 2016). The amino acid COO^−^ and NH_3_^+^ groups may be attracted to Ca^2+^ and CO_3_^2−^ sites on the CaCO_3_ crystal face, as hypothesised in calcite (Kim et al. 2016; Montanari et al. 2016; Wan et al. 2024). Once attached to the crystal surface, the amino acids can become buried by subsequent CaCO_3_ growth (Wan et al. 2024; Cho et al. 2016, 2018). It is unlikely that the amino acid remains at the crystal surface as the CaCO_3_ grows (Cho et al. 2018), as sample cleaning removes all but the intracrystalline amino acids (Penkman et al. 2008). Asp and Glu are structurally similar, with Glu containing one more methylene group in the carboxylic acid side chain. However, the shorter Asp side chain may facilitate binding of the amino acid to adsorption sites (Lemke et al. 2021) and this may explain more efficient incorporation of Asp. Molecular dynamic simulations provide insights into the lattice distortions created by the substitution of Asp and Gly in the calcite lattice (Kim et al. 2016) while density function theory calculations suggest that bonding interactions between pairs of amino acids influence material properties (Wan et al. 2024).

We note that amino acids in biogenic aragonite are predominantly hosted in proteins and peptides (e.g., Penkman et al. 2008) so further work is required to identify how these larger molecules are accommodated into CaCO_3_ mineral structures and influence their material properties.

## 5 Conclusions

The collective observations of this study show that amino acid incorporation likely influences aragonite hardness via multiple mechanisms: changing grain sizes and boundaries on the micron scale, to dislocations on the atomic scale. Precipitation under inferred conditions at the calcification sites of marine organisms generates polycrystalline aragonite particles (Castillo Alvarez et al. 2024). Glycine has the strongest impact on Vickers hardness, likely due to its similarly smaller grain sizes and lattice distortion on atomic scale. In Asp‐bearing aragonite, grain sizes are relatively large compared to grains in the no amino acid control and other amino acid experiments (Figure 3n), which should lead to decreased hardness. Instead, Vickers hardness in Asp‐bearing aragonite is higher than in the control and which could be due to an alternate mechanism, lattice distortions on the atomic scale which likely improve hardness by limiting slip deformation and twinning (Liu et al. 2017)., both of which have been observed in aragonite (San et al. 2022). Grain sizes in Gly‐bearing aragonite are smaller, which improves the resilience of the material to indentation. Lattice distortions of shorter *a*‐axis lengths are observed for increasing Gly and Asp concentrations in aragonite. Glu incorporation also reduces aragonite grain size, but Glu‐bearing aragonite does not attain the same hardness of Gly‐bearing analogs. Incorporation of comparable amounts of Glu and Asp in aragonite has similar effects on hardness, although the mechanisms of hardening are inferred to be different between the two amino acids. We suggest future studies explore the impact of mixed amino acids, lipids, and coral‐associated proteins on the Vicker's hardness and crystallography of precipitates that serve as even closer analogs to coral skeletons.

## Funding

This work was supported by the Natural Environment Research Council (NE/S001417/1).

## Conflicts of Interest

The authors declare no conflicts of interest.

## Supporting information


**Figure S1:** Scanning electron micrographs of (a) a cross‐section through an aragonite particle precipitated with 5 mM aspartic acid before etching and (b) after etching.
**Figure S2:** Representative XRD patterns of all precipitated aragonite using Mo Ka radiation (k = 0.71069 A˚). Crystallographic measurements taken by micro‐powder X‐ray diffraction (XRD) confirm that all the samples measured are aragonite.
**Table S1:** Aragonite [amino acid] in the aragonite samples precipitated with varying seawater [amino acid] or no amino acid (control).
**Table S2:** Vickers hardness (GPa) of aragonite particles in populations precipitated from seawater with no amino acid (control) or with 100 μM and 5 mM of each amino acid.
**Table S3:** Lattice parameters of aragonite precipitated with varying seawater [amino acid]. 5 or 6 replicates were analysed for each population.
**Table S4:** Dunn's post hoc summary (using PAST software) comparing different unit cell parameters of the different experiment. *p*‐values of less than 0.05 are indicate that the groups are statistically significant.
**Figure S3:** Representative Raman spectra of precipitated aragonite without amino acid (control) and with 5 mM amino acid concentrations. Each precipitate and found that lattice mode peaks at 100–250 cm−1 in all spectra were consistent with aragonite with no evidence of the features associated with calcite or amorphous calcium carbonate (Fang et al. 2023).

## Data Availability

The data that support the findings of this study are available in the Supporting Information of this article.
