# Supplementary material for: Origins of Improved Hardness in Bioinspired Aragonite Precipitated Under Simulated Biologic Conditions
Source: Geobiology. 2026 Sep 10;24(5):e70060. doi: 10.1111/gbi.70060 (PMC13560609; doi:10.1111/gbi.70060)
Supplement: Supplementary file 1 [file GBI-24-e70060-s001.docx]

**Origins of improved hardness in bioinspired aragonite precipitated under simulated biologic conditions**

Supplementary Figure S1. Scanning electron micrographs of a) a cross-section through an aragonite particle precipitated with 5 mM aspartic acid before etching and b) after etching.

| a)   | b)   |
| --- | --- |

**Origins of improved hardness in bioinspired aragonite precipitated under simulated biologic conditions**

Supplementary Figure S2. Representative XRD patterns of all precipitated aragonite using Mo Ka radiation (k = 0.71069 A˚). Crystallographic measurements taken by micro-powder X-ray diffraction (XRD) confirm that all the samples measured are aragonite.

**Origins of improved hardness in bioinspired aragonite precipitated under simulated biologic conditions**

Table S1. Aragonite [amino acid] in the aragonite samples precipitated with varying seawater [amino acid] or no amino acid (control).

|  | Seawater [amino acid] µM | Aragonite [amino acid] nmol/mg | | |
| --- | --- | --- | --- | --- |
|  |  | Asp | Gly | Glu |
| This study | 0 | 0.01 | 0.02 | 0.00 |
|  | 1 | 0.07 | 0.04 | 0.00 |
|  | 100 | 1.17 | 0.13 | 0.27 |
|  | 5000 | 25.60 | 2.44 | 3.66 |
| Gardella et al., 2024 | 2000 | 13.11 | 1.25 |  |
|  | 1 | 0.00 |  |  |
|  | 10 | 0.08 |  |  |
|  | 100 | 0.77 |  |  |
|  | 1000 | 5.68 |  |  |

**Origins of improved hardness in bioinspired aragonite precipitated under simulated biologic conditions**

Table S2. Vickers hardness (GPa) of aragonite particles in populations precipitated from seawater with no amino acid (control) or with 100 µM and 5 mM of each amino acid.

| Control | 100 µM Asp | 5 mM Asp | 100 µM Glu | | 5 mM Glu | | 100 µM Gly | 5 mM Gly |
| --- | --- | --- | --- | --- | --- | --- | --- | --- |
| 0.92 | 2.80 | 1.27 | 1.55 | | 1.71 | | 3.38 | 7.77 |
| 0.65 | 1.39 | 1.30 | 1.48 | | 2.82 | | 1.43 | 4.92 |
| 0.75 | 3.05 | 1.23 | 2.26 | | 1.43 | | 1.60 | 3.69 |
| 0.62 | 2.03 | 1.97 | 1.85 | | 3.18 | | 3.47 | 2.82 |
| 0.58 | 1.54 | 4.92 | 2.04 | | 1.36 | | 4.80 | 1.89 |
| 0.88 | 3.27 | 1.61 | 1.91 | | 2.51 | | 2.35 | 5.30 |
| 1.08 | 2.96 | 1.81 | 1.42 | | 2.79 | | 3.28 | 1.32 |
| 0.96 | 2.87 | 7.38 | 2.35 | | 1.88 | | 3.87 | 1.84 |
| 1.04 | 3.31 | 1.61 | 2.71 | | 2.59 | | 3.17 | 2.07 |
| 0.92 | 2.07 | 7.04 | 2.28 | | 3.32 | | 1.73 | 2.72 |
|  | 1.39 |  | 1.88 | | 2.82 | | 2.59 | 6.77 |
|  | 2.79 |  | 2.39 | | 2.68 | | 2.12 |  |
|  | 1.71 |  | 1.99 | | 3.06 | | 1.31 |  |
|  | 2.60 |  | 2.66 | | 2.36 | | 2.88 |  |
|  | 3.19 |  | 2.23 | | 2.36 | |  |  |
|  | 2.67 |  | |  | | 2.88 |  |  |

**Origins of improved hardness in bioinspired aragonite precipitated under simulated biologic conditions**

Table S3. Lattice parameters of aragonite precipitated with varying seawater [amino acid]. 5 or 6 replicates were analysed for each population.

| Seawater [amino acid] | | | | | | | |
| --- | --- | --- | --- | --- | --- | --- | --- |
| 0 | 100 µM Asp | 5 mM Asp | | 100 µM Glu | 5 mM Glu | 100 µM Gly | 5 mM Gly |
| *a*-axis length (Å) | | |  |  |  |  |  |
| 4.9629 | 4.9632 | | 4.9632 | 4.9635 | 4.9631 | 4.9636 | 4.9632 |
| 4.9633 | 4.9637 | | 4.9631 | 4.9638 | 4.9638 | 4.9634 | 4.9623 |
| 4.9633 | 4.9630 | | 4.9622 | 4.9632 | 4.9634 | 4.9651 | 4.9631 |
| 4.9627 | 4.9636 | | 4.9622 | 4.9634 | 4.9632 | 4.9637 | 4.9628 |
| 4.9637 | 4.9637 | | 4.9627 | 4.9639 | 4.9635 | 4.9644 | 4.9631 |
|  | 4.9638 | | 4.9628 | 4.9629 | 4.9637 | 4.9644 | 4.9626 |
| *b*-axis length (Å) | | |  |  |  |  |  |
| 7.9708 | 7.9710 | | 7.9748 | 7.9715 | 7.9713 | 7.9712 | 7.9719 |
| 7.9713 | 7.9715 | | 7.9753 | 7.9721 | 7.9725 | 7.9709 | 7.9712 |
| 7.9710 | 7.9706 | | 7.9736 | 7.9712 | 7.9720 | 7.9738 | 7.9717 |
| 7.9705 | 7.9715 | | 7.9734 | 7.9716 | 7.9718 | 7.9724 | 7.9713 |
| 7.9716 | 7.9718 | | 7.9746 | 7.9719 | 7.9727 | 7.9726 | 7.9716 |
|  | 7.9714 | | 7.9745 | 7.9705 | 7.9729 | 7.9730 | 7.9706 |
| *c*-axis length (Å) | | |  |  |  |  |  |
| 5.7501 | 5.7507 | | 5.7501 | 5.7513 | 5.7500 | 5.7504 | 5.7498 |
| 5.7507 | 5.7508 | | 5.7508 | 5.7503 | 5.7503 | 5.7502 | 5.7485 |
| 5.7505 | 5.7506 | | 5.7495 | 5.7507 | 5.7507 | 5.7521 | 5.7493 |
| 5.7498 | 5.7515 | | 5.7495 | 5.7514 | 5.7502 | 5.7510 | 5.7494 |
| 5.7508 | 5.7510 | | 5.7501 | 5.7522 | 5.7506 | 5.7515 | 5.7497 |
|  | 5.7511 | | 5.7503 | 5.7503 | 5.7508 | 5.7515 | 5.7489 |
| Unit cell volume (Å^3^) | | |  |  |  |  |  |
| 227.47 | 227.51 | | 227.59 | 227.56 | 227.48 | 227.52 | 227.50 |
| 227.52 | 227.55 | | 227.63 | 227.55 | 227.56 | 227.50 | 227.39 |
| 227.50 | 227.48 | | 227.49 | 227.51 | 227.54 | 227.73 | 227.46 |
| 227.44 | 227.57 | | 227.48 | 227.56 | 227.51 | 227.58 | 227.45 |
| 227.55 | 227.57 | | 227.56 | 227.63 | 227.57 | 227.64 | 227.48 |
|  | 227.56 | | 227.58 | 227.46 | 227.59 | 227.65 | 227.40 |

**Origins of improved hardness in bioinspired aragonite precipitated under simulated biologic conditions**

Table S4. Dunn’s post hoc summary (using PAST software) comparing different unit cell parameters of the different experiment. P-values of less than 0.05 are indicate that the groups are statistically significant.

| Sample and number of measurements | p-value | | | |
| --- | --- | --- | --- | --- |
|  | *a*-axis | *b*-axis | *c*-axis | Volume |
| Control (n=5) vs 5mM of aspartic acid (n=6)  Control (n=5) vs 5mM of glutamic acid (n=6)  Control (n=5) vs 5mM of glycine (n=6) | 0.25  0.33  0.26 | **7.2x10^-5^**  **0.019**  0.38 | 0.44  0.93  **0.038** | 0.083  0.16  0.31 |
| 5mM of aspartic acid vs 5mM of glutamic acid (n=6)  5mM of aspartic acid vs 5mM of glycine (n=6)  5mM of glutamic acid vs 5mM of glycine (n=6) | **0.013**  0.77  **0.028** | 0.089  **0.001**  0.12 | 0.37  0.17  **0.023** | 0.73  **0.0040**  0.923 |
| Control (n=5) vs 100µM of aspartic acid (n=6)  Control (n=5) vs 100µM of glutamic acid (n=6)  Control (n=5) vs 100µM of glycine (n=6) | 0.25  0.31  **0.026** | 0.53  0.29  **0.037** | 0.12  0.19  0.12 | 0.18  0.19  **0.014** |
| 100µM of aspartic acid vs 100µM of glutamic acid (n=6)  100µM of aspartic acid vs 100µM of glycine (n=6)  100µM of glutamic acid vs 100µM of glycine (n=6) | 0.89  0.25  0.20 | 0.66  0.13  0.28 | 0.81  0.98  0.80 | 0.97  0.24  **0.015** |
| 5mM of aspartic acid vs 100µM of aspartic acid (n=6)  5mM of glutamic acid vs 100µM of glutamic acid (n=6)  5mM of glycine vs 100µM of glycine (n=6) | **0.0079**  0.96  **0.00045** | **0.00045**  0.17  0.20 | **0.016**  0.20  **0.00014** | 0.69  0.92  **0.00026** |

**Origins of improved hardness in bioinspired aragonite precipitated under simulated biologic conditions**

Supplementary Figure S3. Representative Raman spectra of precipitated aragonite without amino acid (control) and with 5 mM amino acid concentrations. Each precipitate and found that lattice mode peaks at 100–250 cm^-1^ in all spectra were consistent with aragonite with no evidence of the features associated with calcite or amorphous calcium carbonate [55].
